# Clinical and sociodemographic profile of acute intoxications in an emergency department: A retrospective cross-sectional study

**DOI:** 10.3389/fpubh.2022.990262

**Published:** 2022-10-13

**Authors:** Juan José Aguilón-Leiva, Clara Isabel Tejada-Garrido, Emmanuel Echániz-Serrano, Eduardo Mir-Ramos, Antonio Manuel Torres-Pérez, Alberto Lafuente-Jiménez, María Martínez-Soriano, Iván Santolalla-Arnedo, Michal Czapla, Jacek Smereka, Raúl Juárez-Vela, Pedro José Satústegui-Dordá

**Affiliations:** ^1^Aragón Health Service, Hospital of Alcañiz, Alcañiz, Spain; ^2^Group in Research in Care (GRUPAC), Department of Nursing, University of La Rioja, Logrono, Spain; ^3^Research Group of the Transfercult, Department of Physiatry and Nursing, Faculty of Health Sciences, University of Zaragoza, Zaragoza, Spain; ^4^Health Emergencies 061 ARAGÓN, Aragon Health Service, Zaragoza, Spain; ^5^Rioja Health Service, Logrono, Spain; ^6^Department of Emergency Medical Service, Wroclaw Medical University, 51-618 Wroclaw, Poland; ^7^Research Group Water and Environmental Health (B43_20R), Department of Physiatry and Nursing, Faculty of Health Sciences, University of Zaragoza, Zaragoza, Spain

**Keywords:** poisoning, drugs, substance-related disorders, emergency department (ED), intoxications

## Abstract

**Background:**

Epidemiological studies about acute poisoning are useful for developing clinical toxicology, especially those carried out in hospital emergency departments. We aimed to evaluate acute intoxication clinical and sociodemographic profile in South Aragon Hospital, Spain.

**Methods:**

We carried out a retrospective cross-sectional study. We included 442 patients treated for acute poisoning in the emergency department during the 3 years 2015–2018. In the inferential analysis, the Chi-square test was used to compare proportions, and the Mann-Whitney *U*-test was used to compare ranges. A confidence level of 95 per cent was considered in all tests.

**Results:**

The mean age was 44.1 years. 57.2% were men. Drugs of abuse were present in 243 patients (55%), drugs in 172 (38.9%), chemicals in 57 (12.9%) and three patients (0.7%) were poisoned by mushrooms. Nine different drugs of abuse, 73 drugs, 15 chemical compounds and 2 varieties of mushrooms were registered. Of the intoxicated patients, 92.3% had symptoms, 84.2% received treatment and 78.7% were discharged from the emergency department.

**Conclusions:**

We obtain a clear clinical and sociodemographic profile of intoxicated patients who come to the emergency department; the five toxins that cause most acute poisoning are: alcohol, benzodiazepines, antiarrhythmics, cannabis and carbon monoxide.

## Introduction

Knowing the clinical profile of Acute Poisoning (AP) is essential for the correct diagnosis and treatment of medical emergencies (1, 2). Any substance can be poisonous if used incorrectly, in the wrong amount or by the wrong person (2–4). This means that the products involved in poisoning can be very varied. Consultations recorded in hospital emergency services (ES) reveal that drugs of abuse and pharmaceuticals are the most common tox. Although to a lesser extent, exposures to chemical substances from the home, industry or agriculture are also observed, and some infrequent poisonings such as those caused by plants, mushrooms, snake bites, jellyfish stings and other poisonous animals (4–6). Poisoning accounts for around 1% of all hospital emergencies, which is a common clinical situation in these services (7). In addition, it is considered the ideal place to study intoxicated patients, although it can sometimes be complex (2, 4, 8). The assessment of these patients can be affected by factors such as the patient's level of consciousness, their willingness to collaborate, the type of toxic agent and the availability of complementary tests, among others (4).

Toxicological studies in Spain are scarce, even more so when referring to centers located in small cities. Most studies do not include intoxicated patients treated in regional hospitals since their records usually correspond to second and third-level hospitals (9, 10). For this reason, the research that provides data on these emergencies in hospital centers with a lower level of care receives the recognition of experts since they offer another vision of AP (11–13).

For all the reasons stated, the objective of this study was to establish the clinical and sociodemographic profile of patients treated for acute poisoning in the emergency department of the Hospital of Alcañiz, Bajo Aragón, Spain.

## Materials and methods

### Study design and setting

We performed a Retrospective Cross-Sectional Study of acute poisoning treated in the emergency department of the Hospital of Alcañiz. This first-level center is located in the south of the autonomous community of Aragón. Access to information and data collection was divided into two phases: The first part consisted of specifying the AP episodes treated in the emergency department during 2015–2018. Identifying the diagnostic codes related to poisoning in the International Classification of Diseases (9th edition) was necessary. This list was provided to the Hospital Management service, and a list of cases organized by months and years was obtained. The second phase of the work was based on the systematic reading of medical records. It was carried out through the Hospital Clinic Post computer program installed in the hospital's emergency service and the Electronic Medical Record of Aragon, accessed through the Intranet portal of the Aragon Health Service. Consulting these sources allowed access to emergency discharge reports, toxicological analytical results, medical evolution and registered nurses in care. During the review process, the episodes that served the purpose of the work were selected.

The variables were divided into four groups: sociodemographic, clinical, therapeutic and outcome variables. Likewise, depending on the toxic agent involved, the type of intoxication could be medication, drugs of abuse, chemical products (domestic, industrial and agricultural), mushrooms and poisonous animals.

### Study population

**Figure 1:**
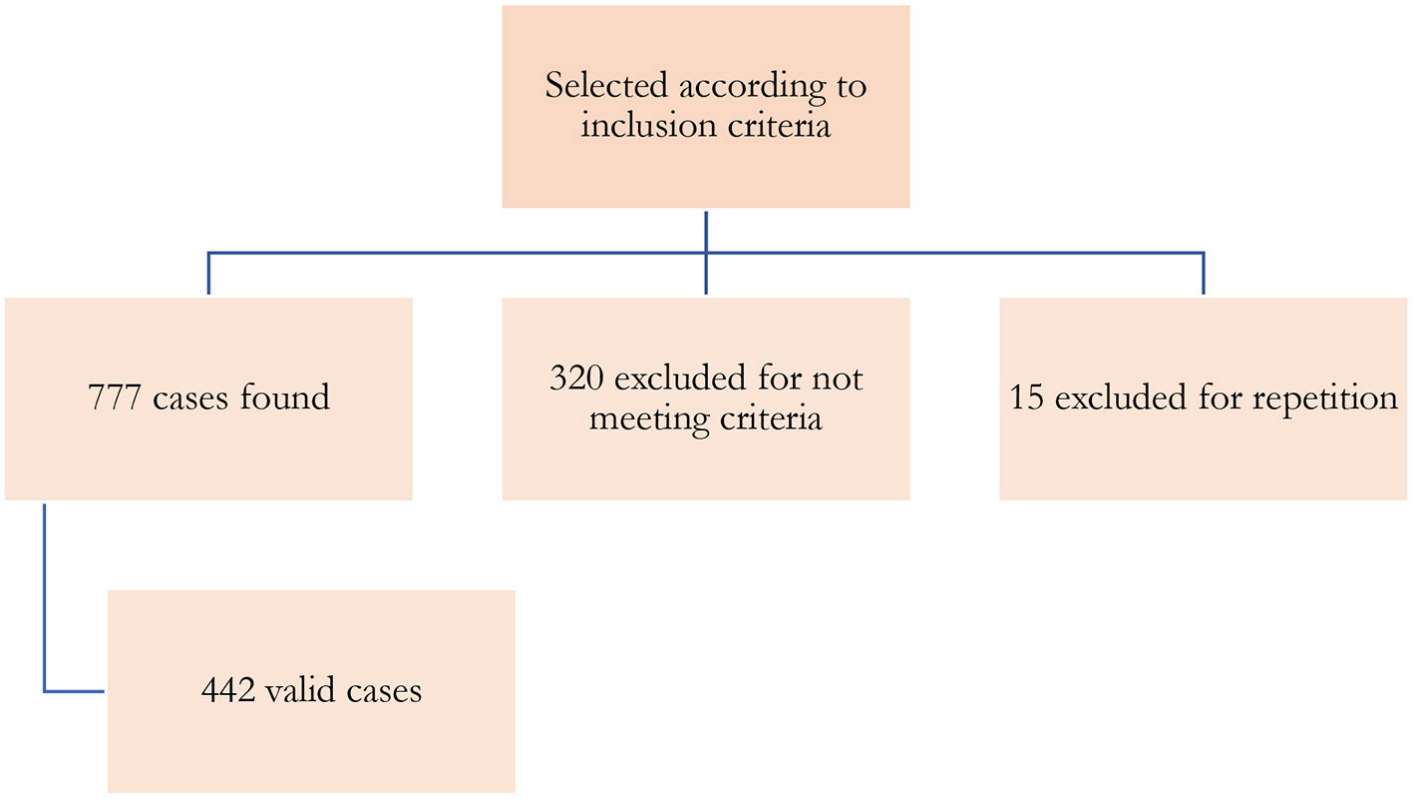
Flow chart.

We analyzed all patients who met the inclusion criteria: Patients of any age with a diagnosis of acute intoxication and in which at least one of these assumptions is met: The discharge report includes one or more coded diagnoses of acute intoxication. The emergency discharge report explains that the reason for care and/or the patient's clinical symptoms are due to recent contact with a toxic substance. The emergency discharge report includes a laboratory determination demonstrating intoxication or Emergency visits derived from an accidental pharmacological overdose and/or a toxic effect on an organ or system. We exclude the Emergency visits derived from a drug's allergic, immunological or idiosyncratic effect. Food poisoning. Chronic poisoning. Insect bites. Ingestion of inert bodies. Exitus due to the toxic cause. Finally, the medical records of patients were examined, of which 15 were repeated, 320 were rejected, and finally, 442 were selected (Figure 1).

### Ethical considerations

This study was approved by the Clinical Research Ethics Committee of Aragón (Act No. 12/2017). The study was carried out in accordance with the tenets of the Declaration of Helsinki and recommendations of good clinical practice. For reporting, the Strengthening the Reporting of Observational Studies in Epidemiology (STROBE) guidelines were followed.

### Statistical analyzes

The SPSS© V25.0 (New York, United States) program was used for the statistical analysis of the data. The distributions of the quantitative variables were summarized with means, standard deviations, medians, and quartiles, whereas the distributions of the qualitative variables were summarized with the number and percentage of occurrence for each of their values. The chi-squared test was used to compare the qualitative variables of the groups. In the case of low values in the contingency tables, the Fisher's exact test was applied instead. The Mann–Whitney test served to compare the quantitative variables of two groups, while the Kruskal–Wallis test (followed by Dunn's *post-hoc* test) was used for comparisons between more than two groups. The relationship between two quantitative variables was assessed using Spearman's correlation coefficient. Linear regressions were used to analyze the impact of potential predictors on the quantitative variables. Regression parameters with 95% confidence intervals were shown.

## Results

The mean age was 44.1 years (SD ± 22.8 years). The youngest patient was ten months old, and the oldest was 96 years old. Between 15 and 55 years of age, 67% of poisonings occurred. 57.2% of the cases belonged to the male sex. There were no differences in age with respect to sex (Table 1).

**Table 1:** Clinical and sociodemographic profile.

**Type of poisoning**	**Toxics**	**n (%)**	**Sex**			**Age groups (years)**						
			**Men**	**Women**	***p***	**Infants and preschoolers**	**School children**	**Teenagers**	**Young adults**	**Old adult**	**>65 years**	***p***
						(≤ 5)	(6–12)	(13–19)	(20–40)	(41–65)	(>65)	
Drugs of abuse	Ethyl alcohol	201 (68.8)	141 (84.9)	60 (77.9)	ns	0 (0.0)	1 (100)	35 (87.5)	74 (74.7)	77 (86.5)	14 (100)	ns
	Cannabis	42 (14.4)	27 (16.3)	15 (19.5)	ns	0 (0.0)	0 (0.0)	7 (17.5)	28 (28.3)	7 (7.9)	0 (0.0)	ns
	Cocaine	33 (11.3)	23 (13.9)	10 (13.0)	ns	0 (0.0)	0 (0.0)	2 (5.0)	18 (18.2)	13 (14.6)	0 (0.0)	ns
	Amphetamines	9 (3.0)	7 (4.2)	2 (2.6)	ns	0 (0.0)	0 (0.0)	0 (0.0)	7 (7.1)	2 (2.2)	0 (0.0)	–
	GHB	2 (0.7)	2 (1.2)	0 (0.0)	–	0 (0.0)	0 (0.0)	1 (2.5)	1 (1.0)	0 (0.0)	0 (0.0)	–
	Total	292 (55)	166 (65.6)	77 (40.7)	<0.001	0 (0.0)	1 (16.7)	40 (78.4)	99 (70.2)	89 (63.1)	14 (15.2)	<0.001
Medicinal drugs	Benzodiazepines	84 (48.8)	31 (44.9)	53 (51.5)	ns	0 (0.0)	0 (0.0)	7 (70.0)	24 (66.7)	37 (72.5)	16 (23.5)	ns
	Antiarrhythmics	44 (25.6)	19 (27.5)	25 (24.3)	ns	0 (0.0)	0 (0.0)	0 (0.0)	0 (0.0)	3 (5.9)	41 (60.3)	ns
	Antidepressants	28 (16.3)	8 (11.6)	20 (19.4)	ns	0 (0.0)	0 (0.0)	3 (30.0)	9 (25.0)	11 (21.6)	5 (7.4)	ns
	Analgesics	18 (10.5)	4 (5.8)	14 (13.6)	ns	1 (20.0)	0 (0.0)	1 (10.0)	5 (13.9)	7 (13.7)	4 (5.9)	ns
	Antipsychotics	18 (10.5)	6 (8.7)	12 (11.7)	ns	0 (0.0)	0 (0.0)	0 (0.0)	9 (25.0)	5 (9.8)	4 (5.9)	ns
	Antiepileptics	18 (10.5)	8 (11.6)	10 (9.7)	ns	0 (0.0)	0 (0.0)	1 (10.0)	4 (11.1)	12 (23.5)	1 (1.5)	ns
	Total	172 (38.9)	69 (27.3)	103 (54.5)	<0.001	5 (50.0)	2 (33.3)	10 (19.6)	36 (25.5)	51 (36.2)	68 (73.9)	<0.001
Chemical products	Carbon monoxide	31 (54.4)	18 (60.0)	13 (48.1)	ns	3 (60.0)	2 (66.7)	0 (0.0)	12 (70.6)	10 (52.6)	4 (40.0)	ns
	chlorine gas	9 (15.5)	3 (10.0)	6 (22.2)	ns	0 (0.0)	0 (0.0)	0 (0.0)	0 (0.0)	5 (26.3)	4 (40.0)	ns
	Bleach	4 (6.9)	1 (3.3)	3 (11.1)	ns	0 (0.0)	1 (33.3)	1 (50.0)	1 (5.9)	0 (0.0)	0 (0.0)	ns
	Total	57 (12.9)	30 (11.9)	27 (14.3)	ns	5 (50.0)	3 (50.0)	2 (3.9)	17 (12.1)	19 (13.5)	10 (10.9)	<0.001
Mushrooms	Total	3 (0.7)	3 (1.2)	0 (0.0)	ns	0 (0.0)	0 (0.0)	0 (0.0)	1 (0.7)	1 (0.7)	1 (1.1)	–

ns, no significance.

**Figure 2:**
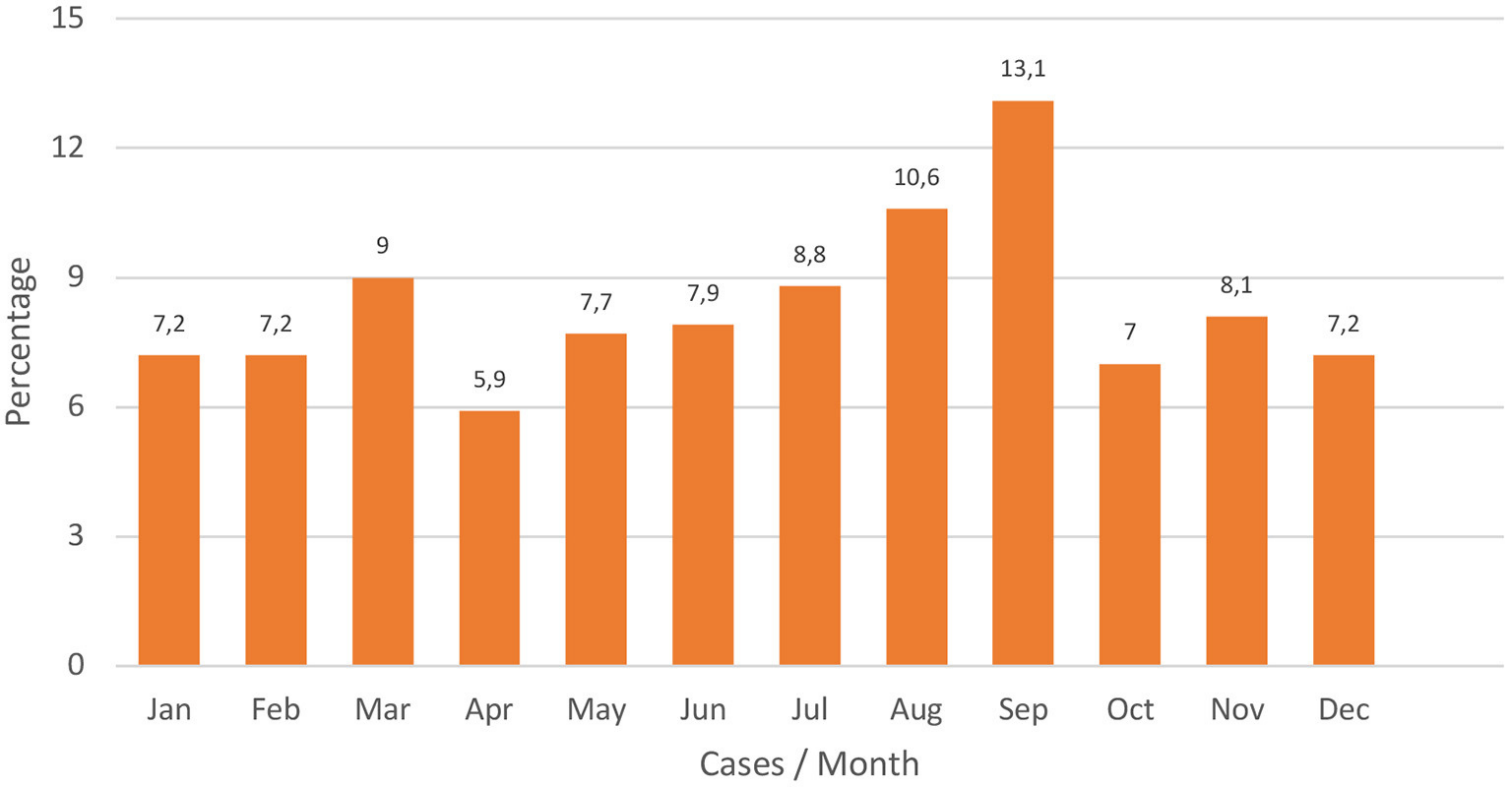
Distribution of cases by months (in percentage).

During the summer period, 29.9% of poisonings occurred, being statistically significant concerning the other times of the year (*p* < 0.05). In the distribution by months, September was the most prominent in a number of cases (*p* < 0.05; Figure 2).

**Figure 3:**
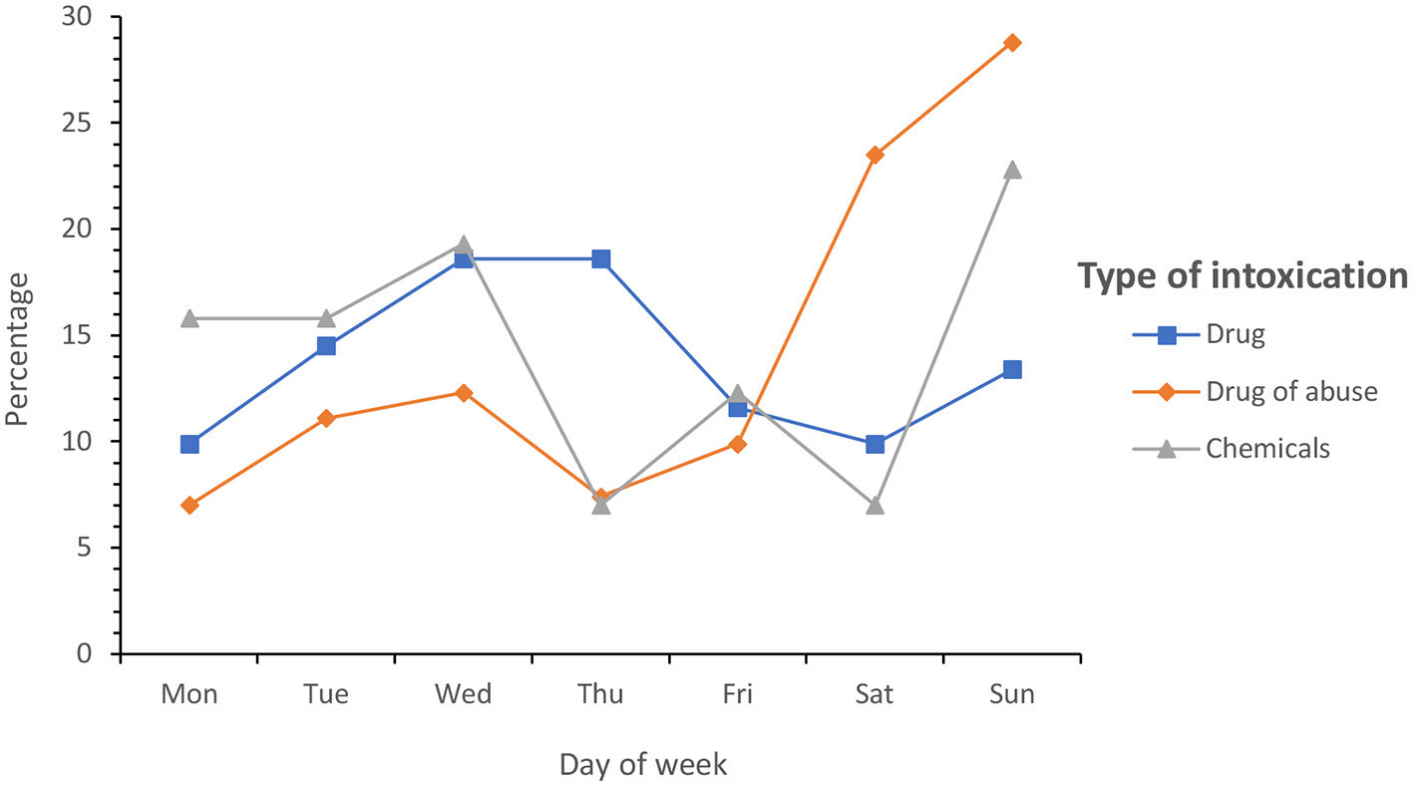
Percentage of cases by type of intoxication and day of the week.

In the analysis of the days of the week, Saturdays (17.6%) and Sundays (22.4%) were the days with the highest incidence. Taking into account the type of intoxication, the following relationship could be established: drug intoxications were more associated with the section of the week from Monday to Thursday and drugs of abuse with weekly fines, from Friday to Sunday (*p* < 0.05). Figure 3 reflects the percentage of cases by type of intoxication and day of the week.

Drugs of abuse were present in 243 patients (55%), medications in 172 (38.9%), chemical products in 57 (12.9%) and only three patients (0.7%) were poisoned by mushrooms (Table 2). No cases related to poisonous animals were found. Nine different drugs, 73 drugs, 15 chemical compounds and 2 varieties of mushrooms were registered. Polyconsumption was not common. 84.4% of the patients took a single drug, and 67.4% a single medication.

**Table 2:** Differentiates the 11 types of AI found in the study (pure and mixed) and their relationship with the patient's fate after consulting the emergency room.

**Types of acute poisoning**	***n***	**Destination of the patient after consulting the emergency room**						***p***
		**Hospital discharge**	**Voluntary discharge**	**Leak**	**Admission to the medical plant**	**ICU admission**	**Admission to Psychiatry**	
Alcohol only	155	138 (89.0%)	6 (3.9%)	5 (3.2%)	3 (1.9%)	0 (0.0%)	3 (1.9%)	<0.001
Only non-benzodiazepine drug^a^	71	40 (56.3%)	1 (1.4%)	0 (0.0%)	24 (33.8%)	1 (1.4%)	5 (7.0%)	
Chemicals	57	52 (91.2%)	0 (0.0%)	0 (0.0%)	4 (7.0%)	1 (1.8%)	0 (0.0%)	
One or more benzodiazepines	35	26 (74.3%)	2 (5.7%)	0 (0.0%)	3 (8.6%)	0 (0.0%)	4 (11.4%)	
Multidrug^b^	35	20 (57.1%)	0 (0.0%)	0 (0.0%)	5 (14.3%)	4 (11.4%)	6 (17.1%)	
Illegal drugs only	32	25 (78.1%)	0 (0.0%)	1 (3.1%)	1 (3.1%)	0 (0.0%)	5 (15.6%)	
Alcohol + illegal drugs	23	17 (73.9%)	1 (4.3%)	1 (4.3%)	1 (4.3%)	0 (0.0%)	3 (13.0%)	
Alcohol + medication	17	15 (88.2%)	1 (5.9%)	0 (0.0%)	0 (0.0%)	0 (0.0%)	1 (5.9%)	
Illegal drugs + medications	10	8 (80.0%)	0 (0.0%)	1 (10.0%)	0 (0.0%)	0 (0.0%)	1 (10.0%)	
Alcohol + illegal drugs + medications	4	4 (100%)	0 (0.0%)	0 (0.0%)	0 (0.0%)	0 (0.0%)	0 (0.0%)	
Mushrooms^c^	3	3 (100%)	0 (0.0%)	0 (0.0%)	0 (0.0%)	0 (0.0%)	0 (0.0%)	

Admissions to the ward and ICU were mainly due to drug poisoning, while those to Psychiatry were divided between drugs of abuse and pharmaceuticals.

a Includes one case that mixed with alcohol.

b Includes cases that have taken several medications simultaneously (the mixture of benzodiazepines with other drugs may occur).

c Includes a case that mixed drugs of abuse.

A relationship was found between drugs of abuse and male gender and age groups ranging from 13 to 65 years (*p* < 0.001). On the contrary, drug intoxications were more associated with the female sex and those over 65 years of age (p<0.001). 92.3% of the intoxicated symptoms presented. Neurological (79%), digestive (21.3%), cardiovascular (19.2%), respiratory (15.8%) and metabolic (6.1%) manifestations were recorded. The highest score on the Glasgow Coma Scale (15 points) was recorded in 81.9% of the patients and only 3.1% had a Glasgow score of less than or equal to 8 on arrival at the emergency room. 84.2% of the patients received some type of pharmacological treatment. The most frequent symptomatic measures were: intravenous fluid replacement (39.1%), analgesia (14.4%), benzodiazepines (10.2%), antiemetics (10.1%) and oxygen (7.9%). In the case of antidotes, the following stood out: thiamine (26.1%), pyridoxine (24%), glucose (22.4%), and flumazenil (14.6%) and high-flow oxygen (6.2%). 78.7% of the patients were discharged from the emergency room. The consultation was not completed in cases of escape (1.8%) and voluntary discharge (2.9%). The rate of admissions to the hospitalization ward at the Alcañiz Hospital was 9%. Other intoxicated patients required interhospital transfer to be admitted to the Intensive Care Unit (ICU) (1.1%) or to the Psychiatric Short Stay Unit (SCU) (5.5%). The average stay in the emergency room was 6 h and 6 min. 31.7% of emergencies were resolved in the first 2 h, and more than half of the cases (58.6%) before 4 h. The mortality rate in the emergency department was 0% for all types of acute poisoning.

## Discussion

This work has focused on elaborating a clinical and sociodemographic profile of the AP treated in the emergency service in Bajo Aragón. Spain. The results cover a period of 3 years and include patients of all ages. It adds interest to this research that it is the first analysis of intoxications in this region.

The consultation rate is practically identical to that reported by other Emergency Departments (9, 10). This concordance of results strengthens the methodology developed in this work. Since our hospital does not have a Clinical Toxicology Unit in the hospital, it was necessary to create its registry of intoxicated patients based on the diagnostic codes assigned to emergency discharge (7).

In the age analysis, a considerable number of patients over 65 years of age (21.3%) were detected. This has led to a rise in the average age, even more so if other publications are taken into account (5, 9, 10, 14). In this sense, it must be taken into account that the region is one of the Spanish provinces with the highest average age (15).

Regarding the distribution by days of the week, the incidence found in other EP and Primary Care was repeated (10, 16). During the weekends, there was a predominance of drug abuse. In addition, the Bajo Aragón concentrates on its local festivals and the celebration of the MotoGP motorcycle world championship in the month of September, so the consumption of psychoactive substances was widespread on those days. This would explain why September was the month with the highest number of cases.

In the analysis of drugs of abuse, alcohol poisoning occupied the most prominent place, something common even at early ages (6, 9, 10, 14, 17). Alcohol was implicated in 45.5% of the total IAs. Cannabis intoxications also stood out, which were ahead of other drugs such as cocaine and amphetamines. In this way, cannabis was confirmed as the illegal substance that caused the most emergencies in Alcañiz. Something that contrasts with the low perception of risk declared by its consumers (18–20). None of the drugs found was new psychoactive substances (synthetic cannabinoids, synthetic cathinone's, piperazines, etc.). At the moment, very few Spanish hospitals are capable of detecting them (12). Drug intoxications were widely distributed. However, benzodiazepine overdoses are very prominent in Spanish Eds (9, 10). In Alcañiz they have been present in 19% of the queries by Accurate Intoxications (AI). The most outstanding data was the incidence of antiarrhythmic drugs in these poisonings, even more so when compared to other hospitals (9–11, 14). The numerous emergencies caused by these drugs are striking, and that 92.3% of them were concentrated in those over 65 years of age. An aging population may have significantly influenced this result. AIs suffered by the elderly are targeted by many authors due to their epidemiological and preventive interest (21, 22).

Exposures to chemical products had a smaller scope than drugs of abuse and medications. The respiratory route was the preferred form of entry for these toxins. Carbon monoxide (CO), chlorine gas, and bleach completed 3 out of 4 poisonings in this group. Interestingly, no cases were recorded from agricultural products, something that might be expected in a rural setting. CO was the most frequent gas. In most cases, the source of CO was due to the incomplete combustion of stoves and heaters in the domestic environment (23). Our region registers very low temperatures during the winter, which may have favored this type of poisoning (24).

In the region, a total of 31 CO intoxications were treated, of which 7 (22%) presented respiratory symptoms. This data contrasts with the percentage of patients (84.6%) who underwent determination of COHb in arterial blood. It is essential that initiatives such as those proposed by the Spanish Foundation for Clinical Toxicology with the “Actions to avoid in acute patient care” gain visibility and help improve aspects such as this (25, 26). Within the therapeutic framework, support measures were the fundamental pillar in treating acutely intoxicated patients, above measures of decontamination, purification, administration of antidotes or any other procedure. The number of digestive decontaminations performed in this study was high compared to the records published annually by the American Association of Poison Control Centers (AAPCC) (27). Many studies have revealed that the combination of LG and CA is not more effective than the exclusive administration of CA (28, 29). However, in Alcañiz, this association occurred 1 out of 3 times. Both the AAPCC and the European Association of Poison Centers and Clinical Toxicologists establish that the routine use of LG in the emergency room should disappear (29). However, the number of decontaminations that is currently performed in clinical practice is decreasing, due to the risk of the appearance of severe complications, such as bronchial aspiration (26, 29–32). Combined treatment with thiamin, pyridoxine and glucose was very widespread in alcohol poisoning. The first two drugs were administered in 42.3% of these abuses, and the three drugs were given in 29.8%. However, none of them is the antidote for acute alcohol intoxication.

National or international recommendations do not include thiamine in the basic provision of antidotes to be used in the care of the intoxicated. In addition, its systemic administration (intravenous or intramuscular) is not recommended. Its use is only appropriate in patients with a clear profile of chronic alcoholism or apparent signs of nutritional deficiencies when they present alcohol intoxication that requires intravenous glucose to correct hypoglycemia to avoid Wernicke's encephalopathy (3, 4, 33, 34). Since these circumstances are exceptional, it is more than likely that these patients have been overused, unnecessarily exposing them to adverse effects. On the other hand, the characteristics of the hospital itself had an influence on some of the results obtained. An example is the transfer rate to other hospitals (8.6%). Alcañiz does not have an ICU or a Psychiatric CSU, so the most severe patients were transferred to the reference hospitals (35). Assistance to an intoxicated person in a regional hospital can have certain advantages. The patients spent little time in the waiting room, and this favored a high-resolution rate in the first few hours. On the contrary, the space is smaller. Perhaps with an emergency observation area with more than 5 beds, the number of admitted patients would have been lower (12).

Some limitations should be considered in this study. This is a retrospective study that only included patients from a single-center. In addition, the most important limitations have been related to time variables. For example, in most cases the care interval was not recorded, nor was the toxic interval (intake-carbon). This has not allowed a more complete analysis of aspects as notable as the correct indication of digestive decontamination and other quality indicators. Further studies should be carried out.

## Conclusion

According to the results obtained, it is possible to conclude that the acute poisonings most frequently treated in the emergency department are caused by alcohol and benzodiazepines. Carbon monoxide, cannabis and antiarrhythmics also report a good number of cases. Men have more intoxications due to drug abuse and women due to medications. The high number of patients intoxicated by drug abuse demonstrates one of the most relevant problems in the field of public health.

## Data availability statement

The raw data supporting the conclusions of this article will be made available upon request to the JA-L.

## Ethics statement

The work was carried out after obtaining the pertinent authorizations in the hospital itself and with the favorable opinion of the Clinical Research Ethics Committee of Aragón (Act No. 12/2017). The rules that regulate access, protection and confidentiality of patient data to Public Administration personnel were strictly complied with.

## Author contributions

JA-L and MM-S: conceptualization. CT-G: methodology. EE-S: software. EM-R: validation. IS-A: formal analysis. MC: investigation. AT-P: resources. JA-L: data curation and writing—original draft preparation. JA-L, RJ-V, MC, and JS: writing—review and editing. PS-D: visualization and project administration. RJ-V, AL-J, and PS-D: supervision. CT-G and IS-A: funding acquisition. All authors contributed to the article and approved the submitted version.

## Conflict of interest

The authors declare that the research was conducted in the absence of any commercial or financial relationships that could be construed as a potential conflict of interest.

## Publisher's note

All claims expressed in this article are solely those of the authors and do not necessarily represent those of their affiliated organizations, or those of the publisher, the editors and the reviewers. Any product that may be evaluated in this article, or claim that may be made by its manufacturer, is not guaranteed or endorsed by the publisher.
